# Durable Metastatic Melanoma Remission Following Pembrolizumab and Radiotherapy: A Case Report of Prophylactic Immunosuppression in a Patient with Myasthenia Gravis and Immune-Mediated Colitis

**DOI:** 10.3389/fimmu.2021.788499

**Published:** 2021-12-08

**Authors:** Luke A. Moradi, Curtis A. Clark, Craig S. Schneider, Alok S. Deshane, Michael C. Dobelbower

**Affiliations:** ^1^ Department of Radiation Oncology, University of Alabama at Birmingham, Birmingham, AL, United States; ^2^ Case Western Reserve University School of Medicine, Cleveland, OH, United States

**Keywords:** metastatic melanoma, autoimmune myasthenia gravis, radiation therapy, abscopal effect, immunotherapy case report

## Abstract

Immune checkpoint inhibitors (ICIs) and radiotherapy (RT) combinations for various metastatic cancers are increasingly utilized, yet the augmentation of anti-cancer immunity including distant tumor responses by RT remains ill-characterized. Immunosuppressive tumor microenvironments and defective anti-tumor immune activation including immune-related adverse events (irAEs) likely limit dramatic immuno-radiotherapy combinations, though it remains unclear which immune characteristics mediate dramatic systemic tumor regression in only a small subset of patients. Moreover, the efficacy of ICI treatment in patients receiving immunosuppressive therapies for autoimmune conditions or irAEs is convoluted, yet clinically valuable. Here, we report a case of a 75-year-old man with myasthenia gravis and metastatic melanoma who experienced complete and durable systemic regression after receiving pembrolizumab and single-lesion RT while on prednisone for myasthenia gravis prophylaxis and vedolizumab for immune-mediated colitis after previously experiencing mixed response on pembrolizumab monotherapy. We discuss the potential paradoxical effects and clinical considerations of immunosuppressive regimens in patients with underlying autoimmune disease or adverse immune reactions while receiving immuno-radiotherapy combinations.

## Introduction

Immune checkpoint inhibitors (ICIs) have become a mainstay of treatment for metastatic melanoma and can elicit durable responses in a subset of patients. One clinical imitation of ICIs is sporadic, aberrant immune-related adverse events (irAEs), resulting in poor tolerance, therapy cessation, and/or implementation of immunosuppressive regimens. Given that irAEs are relatively common in patients receiving ICIs and especially in individuals with underlying autoimmune conditions (1), the original ICI clinical trials excluded individuals with autoimmune diseases. Although more recent studies have demonstrated safety and efficacy of ICIs in patients with concurrent autoimmune disease, incidence of irAEs requiring immunosuppressive therapy may nevertheless result in permanent ICI discontinuation (1, 2). Altogether, the efficacy of ICIs while on immunosuppressants for autoimmunity and/or irAEs remains a prevalent clinical concern.

Despite ICI successes, the reality is that most patients eventually experience disease progression and acquired resistance among responders is problematic (3, 4), emphasizing the need for refining immuno-oncologic strategies in the metastatic setting. Radiotherapy (RT) is utilized in nearly two-thirds of solid tumor cases to improve local tumor control *via* RT-mediated DNA damage of tumor cells, but also with the added advantage of synergistic anti-tumor immune activation to potentiate ICI therapy and in sporadic instances produce distant presumably immune-mediated tumor rejection (abscopal effect) (2, 5, 6). Combination of ICIs and RT has thus emerged as an exciting dual modality strategy to potentiate ICI therapy and circumvent mechanisms of resistance, with isolated reports of dramatic responses in several tumor types (2, 5, 7, 8). While RT-induced abscopal effects were first described more than 50 years ago (9), complete and durable out-of-field responses are exceptionally rare. Intact anti-tumor immunity likely increases the chance of RT-ICI synergy, which is clinically complicated in patients with baseline autoimmune conditions requiring prophylactic and/or irAE-related immunosuppression. Therefore, increased understanding of the impact autoimmune processes and immunosuppression have on RT-ICI responses would help define clinical indications for RT-ICI use in the setting of autoimmunity and may provide clinically valuable insight into mechanisms of anti-tumor immunity in the radio-immunotherapy setting.

Here, we describe the case of a medically immunosuppressed patient with myasthenia gravis (MG) and metastatic melanoma who experienced complete and durable melanoma remission after receiving combination pembrolizumab plus single lesion RT after previously experiencing incomplete response on ICI monotherapy and immune-mediated colitis. We further discuss possible mechanisms with review of the literature and a focused discussion of abscopal effects of RT concurrent with ICI and the implications of irAEs in patients undergoing ICI therapy, particularly those with concomitant autoimmune disease.

## Case Description

A 75-year-old man with MG and a past medical history of prostate cancer status post prostatectomy in 1998 presented to the oncology clinic in November of 2018 after a chest x-ray from a bicycling accident incidentally revealed multiple lung masses up to 4 cm in size. Biopsies of the lung mass revealed melanoma without BRAF V600 mutations (BRAF wild-type), resulting in the diagnosis of stage IV melanoma with unknown primary. The patient was completely asymptomatic at the time of diagnosis with excellent performance status and was still riding a stationary bike 80 miles per week. He was previously diagnosed with MG in 1997 *via* single fiber EMG and Tensilon test after 5 years of fatigue and positional diplopia. He was initially managed with pyridostigmine, but ultimately required corticosteroids due to poor response and was maintained on 10 mg of prednisone daily prior to his melanoma diagnosis.

**Figure 1:**
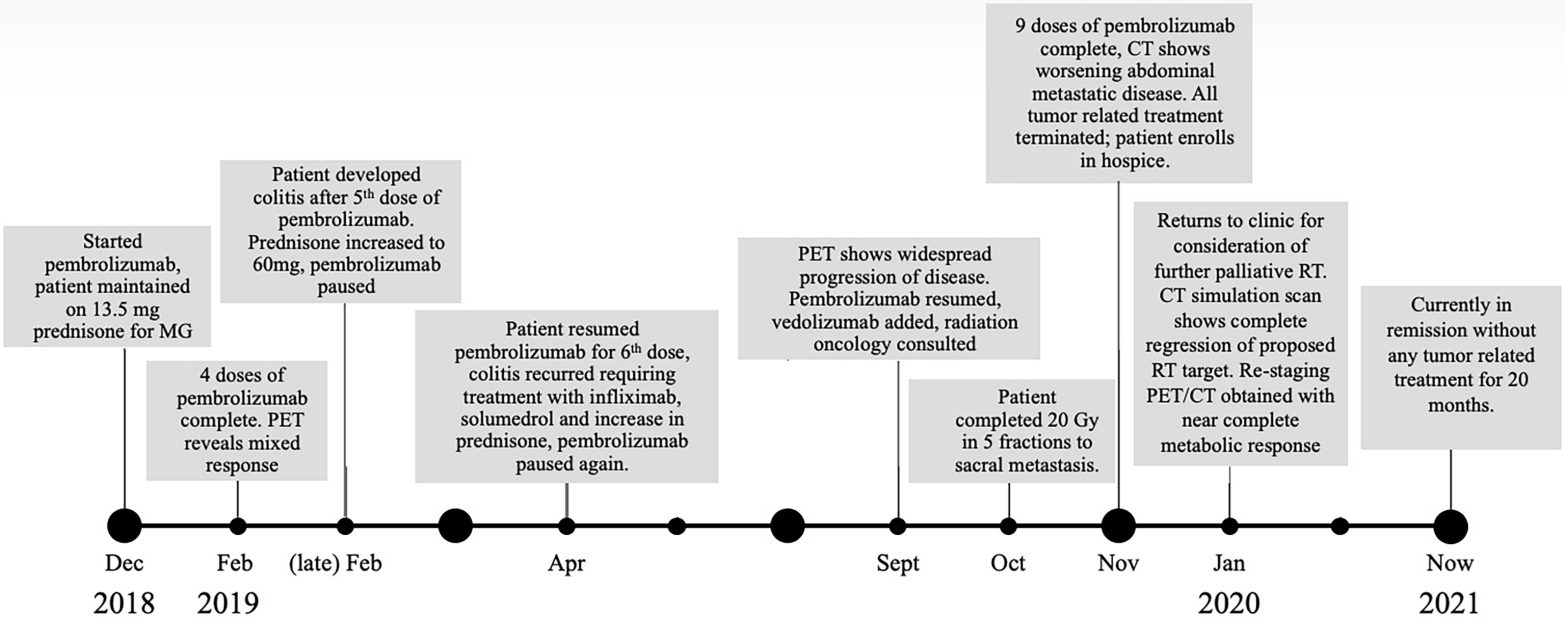
Case timeline detailing therapeutic interventions and clinical responses.

**Figure 2:**
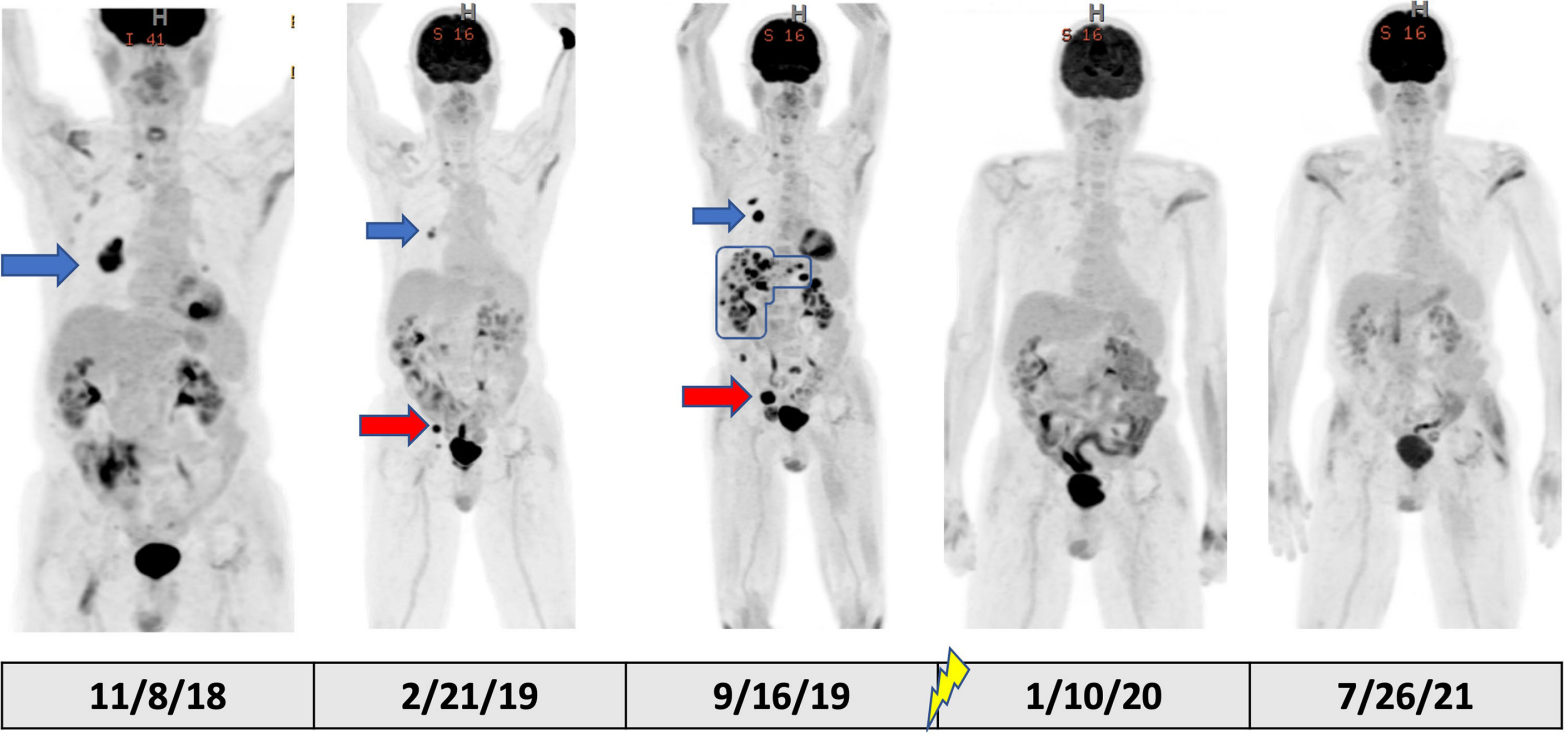
PET/CT scout images over the course of the patient’s treatment. The blue arrows in the figure show the RLL nodule with good response to initial pembrolizumab monotherapy in February 2019 from the baseline scan in November 2018. However, the red arrows demonstrate a site of progression in the right pelvic sidewall through initial pembrolizumab monotherapy representing mixed response to initial pembrolizumab monotherapy. The September 2019 scan, after a break in pembrolizumab therapy for colitis, clearly shows progression of these lesions along with emergency of new, multifocal liver metastases (outlined with blue line). In October 2019, palliative RT was delivered to sacral metastatic lesion (signified by lightning symbol in the timeline). Subsequent PET/CT in January 2020 and July 2021 showed complete and durable response after completion of RT.

**Figure 3:**
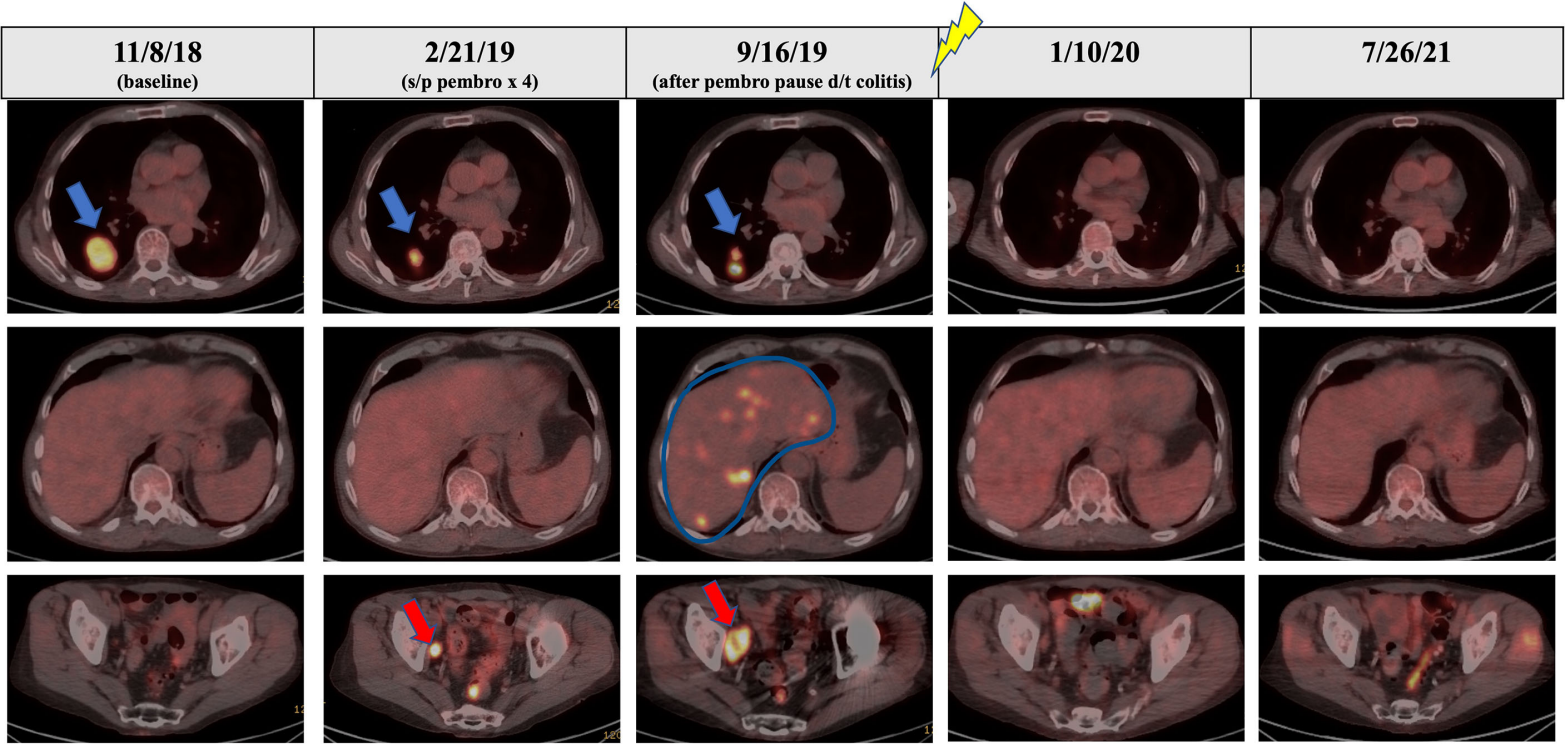
PET/CT scans over the course of the patient’s treatment. The blue arrow in the top panel shows RLL nodule with good response to initial pembrolizumab monotherapy from the baseline scan in November 2018. The red arrow in the bottom panel shows a site of progression in the right pelvic sidewall through initial pembrolizumab monotherapy representing mixed response to initial pembrolizumab monotherapy. Palliative RT was delivered October 2019 to sacral metastasis (signified by lightning symbol). The blue line in the middle panel outlines multifocal progressive disease in the liver seen on the September 2019 PET/CT after break in therapy due to pembrolizumab-related colitis. PET/CT in January 2020 and September 2021 show complete metabolic response.

After his metastatic melanoma diagnosis, pembrolizumab (200 mg at 3-week intervals) monotherapy was initiated in December of 2018 (**Figure 1**). Regarding underlying MG, he was prophylactically maintained on 13.5 mg daily prednisone with the combined goal of MG control and limiting ICI-induced irAEs. After four doses of pembrolizumab, restaging PET imaging in February 2019 demonstrated a mixed response with a 74% decrease in size of his dominant right lower lobe (RLL) nodule (from 4.2 × 3.6 cm to 2.2 × 1.8 cm with SUV decrease 31% from 9.4 to 6.5), a decrease in size of the left lower lobe (LLL) nodule by 80% (from 1.6 × 2.2 cm to 0.9 × 0.8 cm with no residual metabolic activity), but a 33% increase in size of right obturator lymph node (from 1 cm to 1.5 cm with an SUV increase of 83% from 2.1 to 12.2), and new sub-centimeter hypermetabolic pelvic sidewall node (**Figures 2**, **3**). Unfortunately, the patient developed grade 3 colitis secondary to pembrolizumab after his fifth dose, requiring an increase in prednisone to 60 mg daily. Pembrolizumab treatment was paused at this time to allow for resolution of colitis and dose-tapering of prednisone to baseline of 13 mg daily. Pembrolizumab was subsequently resumed in April 2019, but was again discontinued after one dose as the result of active lymphocytic colitis recurrence, this time requiring two doses of infliximab 300 mg, two doses of methylprednisolone 40 mg, and an increase in daily prednisone to 60 mg.

Following a 5-month cessation in melanoma therapy, a PET scan was repeated in September 2019 and revealed numerous new liver metastases, a new sacral metastasis, two new lung nodules, and an increase in PET avidity of RLL nodule by 57% compared to February PET (**Figure 3**). The decision was made to resume pembrolizumab and pretreat with vedolizumab (300 mg at 3-week intervals), a gastrointestinal-specific integrin α4β7 inhibitor, for colitis prophylaxis. All the while, the patient continued taking 12–15 mg of prednisone daily for MG. Radiation oncology was consulted at this time for consideration of palliative radiation to the painful new sacral metastasis. He completed 20 Gy in five fractions over 7 days to the sacral metastasis in mid-October 2019 and received two more doses of pembrolizumab with vedolizumab with the last dose in early November 2019 (**Figure 1**).

Four days following the last pembrolizumab dose, the patient returned for an unscheduled follow-up secondary to weakness, fatigue, and presumed melanoma progression with a new, large palpable epigastric mass consistent with hepatic metastasis. CT chest at the time showed a mixed response, compared to the September PET, with resolution of pleural-based left lower lobe nodule, 57% size increase in RLL nodule compared to September PET, new 1.2 cm RLL lesion, RLL pleural/subpleural thickening, and a new small left upper lobe nodule. CT abdomen revealed significant interval increase in metastatic disease burden with liver involvement, increase in metastatic implants adjacent to the left kidney, and several new sites of abdominal and pelvic disease. For context, the RT-treated sacral lesion demonstrated stable FDG avidity.

At this time (approximately 1 month since completion of RT), the decision was made to discontinue all tumor-directed therapy and the patient was referred to hospice. Of note, the patient continued daily prednisone and never experienced any further symptoms of colitis while on vedolizumab. One month after being placed on hospice, the patient returned to our radiation oncology department for consideration of palliative RT for new-onset right buttock pain and radiculopathy, which was believed to be from a PET positive lymph node adjacent to the sciatic nerve visualized on previous imaging. A CT simulation scan was performed for RT planning and surprisingly revealed complete remission of the proposed lymph node target. As a result of this unexpected finding, a PET scan was ordered for restaging purposes and revealed a near-complete metabolic response with complete resolution of FDG activity in metastatic liver disease, as well as improved pulmonary, pelvic, and sacral metastatic disease (**Figure 3**).

Because of the near-complete response, the decision was made to continue to observe the patient with no further RT or other melanoma-specific therapies. PET scans were repeated every 3 to 4 months which continued to show complete metabolic response to all sites (**Figures 2**, **3**) of systemic disease as well as near-complete radiographic response *via* CT. At the time of this report, the patient is now 2 years out from discontinuing all therapy and is completely asymptomatic from his previously widely metastatic melanoma with no radiographic evidence of disease.

## Discussion

Here, we report a dramatic, complete, and durable response to ICI-RT in the setting of ongoing immunosuppression in a patient with MG experiencing immune-related colitis during prior pembrolizumab monotherapy. This patient experienced an initial mixed response to four doses of pembrolizumab complicated by development of grade 3 irAE during therapy. This mixed response was composed of marked response of multiple lung metastases and progression at several sites including new disease in the pelvis. After development of metastatic disease progression following the interval of pembrolizumab cessation as the result of ICI-related colitis, the addition of vedolizumab and single-site RT to pembrolizumab therapy corresponded to dramatic reductions in distant, out-of-RT-field lesions over the course of approximately 12 weeks. This complete metabolic tumor response has continued for 2 years in the absence of subsequent oncologic therapy.

Few cases of synergistic response from ICIs and single-site RT leading to dramatic widespread tumor regression have been reported in autoimmune patients (8, 10). In a case by Frohne et al., a patient with metastatic melanoma and underlying Crohn’s disease had a durable response after combined pembrolizumab and vedolizumab with single-site RT (8). Like the case presented here, the potential systemic effects of RT were confounded by ICI timing, precluding determination of whether the response should be attributed to ICI alone or ICI + RT. Our case is unique in that our patient was maintained on high-dose corticosteroids throughout the course of pembrolizumab therapy. Additionally, the second trial of pembrolizumab with the addition of RT was under significantly more immune suppression compared to the initial course that produced a mixed response in the setting of a therapy-discontinuing irAE. Together, the present case illustrates a clinically valuable example of ICI treatment response during concurrent use of high-dose immunosuppressives, persistence in ICI use despite irAEs with use of compartmental-specific immunosuppressives, as well as the potential immune augmenting properties of single-site RT following mixed response to ICI-monotherapy.

It is clinically important to highlight the fact that the patient was on no less than 12 mg of daily prednisone for MG prophylaxis throughout his entire course of pembrolizumab treatment. Because of the immunosuppressive nature of corticosteroids, it is not standard of care or immunologically parsimonious to manage patients on ICI with autoimmune disease with physiologic or supraphysiologic doses of corticosteroids. In fact, immunosuppressives including corticosteroids are relatively contraindicated during ICI therapy given the negative impact on anti-tumor immunity and ICI efficacy. Although the use of prednisone in the context of autoimmunity may have contributed to the initial mixed response to pembrolizumab monotherapy, this patient still developed immune-related colitis, leading to treatment hiatus, further increase in steroid dose, and eventual widespread tumor progression. The relatively short trial of pembrolizumab prior to treatment cessation may also have contributed to poor response; however, it is important to note development of new tumors while on pembrolizumab monotherapy and prior to RT (**Figures 2**, **3**).

Despite continuation of 12–15 mg of prednisone daily, the patient experienced a dramatic and durable response after resumption of pembrolizumab with the addition of vedolizumab and single-site RT. While it is possible that this dramatic response may be simply attributed to delayed response to pembrolizumab made possible through the gut-protective effects of vedolizumab, it is within reason that an abscopal effect from single-site RT may have provided a catalyst for widespread immune activation. In cases of potential abscopal effects, it is ideal albeit logistically and technically impractical to investigate tumor-infiltrating lymphocytes or peripheral immune activity proximally before and after RT to mechanistically define the immunologic effects of RT, particularly during concurrent immune-augmenting therapy use. Notwithstanding, the continued use of systemic immunosuppressants and prior mixed response to ICI monotherapy in this case supports the likelihood that RT played a significant role in the patient’s dramatic systemic response.

Importantly, the development of ICI-related colitis in the background of pre-existing autoimmune disease in this case highlights significant clinical questions regarding the persistence of ICI use despite irAEs that may be handled potentially with adjuvant therapies (i.e., vedolizumab in this case), which require further exploration of compartmental-specific immunosuppressants. For example, the contribution of α4β7 integrin blockade to immune cell circulation/bioavailability is uncertain in this case; however, it is interesting to speculate that selective trafficking of lymphocyte subpopulations during ICI therapy may be therapeutically exploitable. Moving forward, combination of systemic and compartment-selective immunosuppressing drugs may be crucial in allowing ICI use in autoimmune individuals to prevent anticipated irAEs or autoimmune flares. The contribution of combination ICI-RT in these populations must also be considered as our understanding of abscopal mechanisms advances and the systemic anti-tumor immune milieu required for higher incidence of abscopal effects may have important therapeutic implications, with the potential for broader clinical implications to the general study of immunologic factors driving response and resistance to immunotherapy.

## Conclusion

In this case report, ICI therapy and single-site RT in a patient with MG receiving systemic and tissue-specific immunosuppressants induced a profound systemic treatment response resulting in complete metabolic regression and durable response to his metastatic melanoma. Given the prior mixed response to ICI in this case, as well as continued systemic immunosuppression for MG and irAE prophylaxis, a contributory immune-augmenting abscopal effect from RT remains significantly plausible. Clinically, this case highlights the (1) potential for multi-modality, radio-immunotherapeutic synergy in metastatic malignancies including enhanced abscopal-related effects; (2) potential response to RT-ICI in the context of concurrent autoimmune disease and immunosuppressive regimens (i.e., corticosteroids) with implications that may broaden the utility and understanding of factors that facilitate RT-ICI response in immunodeficient populations; and (3) potential for immune-modifiers (e.g., compartmental-specific immune inhibitors; vedolizumab) to enhance tolerability and continuation of ICIs in the context of irAEs and ongoing systemic immunosuppression.

## Patient’s Perspective

“My journey began with a cycling accident that routine x-rays revealed the lesion(s) in my lung. The following year was characterized by a bunch of ups and downs, response to treatment and no response, better then worse, and on and off Keytruda because of recurrent colitis. It was at best a frustrating year because of those factors. Having read about the use of Entyvio in connection with PD-1 blockers. I discussed it with my oncologist and restarted treatment in September 2019. Although no colitis ensued the metastases dramatically worsened on treatment, so I was put on Hospice; “nothing else we can do.”

From September 2019 until the first of January 2020, I was miserable, constant Intractable nausea, pain, fatigue, weakness, and weight loss. I prepared to die focused on getting all of my affairs on order. It was “melanoma, melanoma, melanoma day-in and day-out. As an ardent believer in the power of prayer, I had numerous prayer groups, relatives and friends offering them up. This sustained me. Death is not to be feared, because you never actually meet it. I “crossed over” so to speak and actually looked forward to the peace and comfort Heaven provides and the reunion with lost loved ones. Then, two months later being told the “cancer is all gone,” needs no elaboration. Elation, gratitude, peace.”

Robbie

## Data Availability Statement

The original contributions presented in the study are included in the article/supplementary material. Further inquiries can be directed to the corresponding author.

## Ethics Statement

Written informed consent was obtained from the individual(s) for the publication of any potentially identifiable images or data included in this article.

## Author Contributions

LM, AD, CS, and CC contributed significantly to the conception and first draft of the case report. CS, LM, CC, and MD contributed significantly to editing and approval of publishing. All authors contributed to the article and approved the submitted version.

## Conflict of Interest

The authors declare that the research was conducted in the absence of any commercial or financial relationships that could be construed as a potential conflict of interest.

## Publisher’s Note

All claims expressed in this article are solely those of the authors and do not necessarily represent those of their affiliated organizations, or those of the publisher, the editors and the reviewers. Any product that may be evaluated in this article, or claim that may be made by its manufacturer, is not guaranteed or endorsed by the publisher.
